# Hypodermic Injections

**Published:** 1875-05

**Authors:** D. McLean Forman

**Affiliations:** Freehold, New Jersey


### HYPODERMIC INJECTIONS.

BY D. McLEAN FORMAN, M. D., FREEHOLD, NEW JERSEY.

My experience in the use of hypodermic injections has, during the past seven years, been quite extensive. While an interne in Bellevue and St. Luke's Hospitals, I seldom made my evening visit to my wards without a hypodermic syringe in my pocket; and as, in all large hospitals, there are a certain number of patients suffering from incurable and painful diseases, the syringe was often called into service to afford them temporary relief from their sufferings. For this class of patients, I believe the hypodermic injection of morphine affords greater relief from pain than can be obtained in any other way; and, in my experience, when thus administered, it is much less likely to produce the unpleasant remote effects, such as nausea, vomiting and constipation, which so often attend the administration of the drug by the mouth.

For the relief of acute pain, I have used it in many instances. In cases of renal and biliary calculi, during the passage of the stone, it has afforded speedy and marked relief from pain.

In intercostal neuralgia, and in the pleuritic pains so common in the advanced stages of phthisis, a few drops of morphine injected at the seat of pain, afford decided relief.

In other forms of neuralgia, its administration, for temporary relief, has been attended by success.

In every civilized community there are a certain number of females who suffer from what may be termed malignant hysteria. In these patients there is no end to their aches and pains, and no region of the body that is exempt from them. In these cases, if, after having exhausted the Materia Medica without affording them relief from their sufferings, the physician, as a last resort, makes use of the hypodermic syringe, he will be surprised to find what speedy relief he gives them; but he need not be surprised when, in a few days, he is again sent for to repeat the operation. The patient has, at last, found something that will remove her pain and, at the same time, produce a pleasant intoxication; and if she does not remove out of the reach of the doctor, her attacks will be frequent and her importunities for the use of the syringe so urgent, as to make him regret the invention of the instrument.

In sciatica its use has been attended by marked success. A single injection along the course of the nerve will often give the patient a comfortable night; and thus, by preventing the exhaustion induced by constant suffering, greatly expedite recovery.

In cholera and cholera morbus, where the stomach is so irritable as to reject everything taken into it, the vomiting and cramps have, in many instances, been promptly checked by a single injection of morphine.

In a case of convulsions, occurring in a patient who had been a long time sick with cerebro-spinal meningitis, the injection of morphine seemed to be of service; also, in a case of a professional friend's, in which the patient had from a dozen to twenty convulsions daily, depending upon an affection of the spinal cord, an injection of morphine, night and morning, reduced the number of convulsions to one or two, daily. Each time the morphia was omitted, the convulsions increased in frequency. In several cases of traumatic tetanus, hypodermic injections diminished the number of convulsions, though the patient ultimately died of that disease.

In three cases of puerperal convulsions, the hypodermic injection of morphine was used. In two of these cases there were no convulsions after the injection was given, but as venesection was also employed immediately before the injection, we cannot say how much was due to its influence. The other case was not benefited by them, the patient continuing to have convulsions until she died.

In two cases of flooding, one ante-partum, one post-partum, large injections of morphine were administered. The stimulating effect of the drug when given in large doses, was immediate and very marked.

In using morphine for hypodermic injection, I always use what is known as Magendie's solution.

For the relief of moderate pains, I rarely administer more than five minims of the solution.

For severe pain or convulsions, I give from fifteen to twenty minims; also about twenty minims after severe hemorrhage.

The point at which the injection is made, (except in cases of sciatica), I think makes but little difference. One patient suffering from elephantiasis of the leg in about a year and a half received nearly one thousand injections in various portions of her cellular tissue. Cases requiring frequent injections are likely to be troubled with abscesses, if contiguous parts are too frequently injected. Abscesses occasionally follow a single injection. Thinking perhaps this might be due to the irritation produced by a fungus which develops in the solution of morphine after it has stood a few weeks, about a year ago I added a grain of carbolic acid to each ounce of the solution. This prevents the growth of the fungus, and the solution remains clear indefinitely. Since using this solution I have had no abscesses.

When medicine is used hypodermically, a much smaller quantity is required to produce the physiological effects of the drug, than when administered in other ways. I do not think that morphia when used in this way is as apt to produce nausea, vomiting and constipation as when given by the mouth. I have several patients who are always sickened by it when given by the mouth; but when given by hypodermic injection, it produces none of these unpleasant effects.

In cases of malarial fever (intermittent, contracted at Panama,) I have used hypodermic injections of quinine in three or four cases. The fever, in each, was cured, but troublesome abscesses occurred at many of the injected points.

In several cases of secondary syphilis I have used calomel in glycerin, as an injection. In these cases nearly every injection was followed by an abscess, and I do not know that this method of treatment possesses any advantage over the one usually adopted.

In three cases of varicose veins of the leg, I have injected a single drop of the liquor ferri persulph, in several of the most prominent points. These operations were entirely successful in curing the disease, and I regard this method as one of the most useful in the treatment of this affection.

